# Lactylation: a Passing Fad or the Future of Posttranslational Modification

**DOI:** 10.1007/s10753-022-01637-w

**Published:** 2022-02-27

**Authors:** Qi Xin, Hai Wang, Qinglin Li, Sinan Liu, Kai Qu, Chang Liu, Jingyao Zhang

**Affiliations:** 1 Department of Hepatobiliary Surgery, The First Affiliated Hospital of Xi’an Jiaotong University, Xi’an, 710061 Shaanxi China; 2 Department of SICU, The First Affiliated Hospital of Xi’an Jiaotong University, Xi’an, 710061 Shaanxi China

**Keywords:** lactylation, lactate, posttranslational modification, gene transcription, cancer, inflammation

## Abstract

Lactate is a glycolytic product and a significant energy source. Moreover, it regulates gene transcription via lactylation of histones and non-histone proteins, i.e., a novel posttranslational modification. This review summarizes recent advances related to lactylation in lactate metabolism and diseases. Notably, lactylation plays a vital role in cancer, inflammation, and regeneration; however, the specific mechanism remains unclear. Histone lactylation regulates oncogenic processes by targeting gene transcription and inflammation via macrophage activation. Eventually, we identified research gaps and recommended several primary directions for further studies.

## INTRODUCTION

Lactate formation due to hypoxia was first recognized in the nineteenth century [1]. Recent studies indicate that lactate is a significant energy source and major gluconeogenic precursor during metabolism and a signaling molecule [2]. Herein, we summarize recent advances in lactate metabolism and lactylation of histones and non-histone proteins.

### Lactate Production with Warburg Effect

As a vital carbon metabolite produced during glycolysis, lactate has been extensively investigated due to the Warburg effect of aerobic glycolysis in cancer. Of note, cancer cells produce lactate and adenosine triphosphate (ATP) via glycolysis even under completely aerobic conditions, a phenomenon termed the Warburg effect [3, 4]. Accumulating studies show that the Warburg effect increases the rate of lactate production and acidify the tumor microenvironment (TME) because of the defective mitochondria and impaired adenosine triphosphate (ATP) production; this benefits cell growth and survival [4]. Lactate is predominantly cleared by the liver through gluconeogenesis (the Cori cycle) and ATP production (oxidative phosphorylation, the Krebs cycle). As a chiral compound, lactate has two enantiomers, i.e., L-lactate and D-lactate. L-lactate is a primary product of glycolysis in mammals and is continuously used in various cells, tissues, and organs. In contrast, D-lactate, primarily produced by glyoxalase to metabolize methylglyoxal, is an atypical mammalian metabolite with low concentration at the nanomolar level in normal cells. Carlos et al. revealed that L-lactate but not D-lactate is used in histone lysine lactylation (Kla) [5].

### Lactate Shuttles via MCTs

Accumulation of lactate in the extracellular fluid causes the loss of bicarbonate anions (HCO3-) and the gain of lactate anions (C3H5O3-), which in turn induces lactic acidosis and aggravates sepsis [6–8]. Moreover, lactate shuttles include both intracellular and cell–cell shuttles, which regulate energy delivery and communication. The intracellular lactate shuttles include the cytosol-mitochondrial and cytosol-peroxisome shuttles, whereas the cell–cell lactate shuttles include lactate exchange between white (glycolytic) and red (oxidative) fibers within a muscle bed and between skeletal muscle and organs [2]. Lactate shuttles are mediated by concentration gradients, pH gradients, and redox states. Lactate is transported across the plasma membrane by several monocarboxylate transporters (MCTs), mainly MCT1 and MCT4 [9]. The primary physiological function of MCT1 is lactate importation, while that of MCT4 is lactate exportation. Additionally, the direction of lactate transfer *in vivo* by MCTs depends on lactate and proton concentration gradients [10]. Therefore, lactate importation into the cell is stimulated by reducing both intracellular lactate concentration and pH gradients. CD147, a cochaperone of MCTs, promotes proper expression and localization of MCT1 and MCT4 at the cell surface [2, 9]. Studies indicate that besides lactate, butyrate, and testosterone, thyroid-stimulating hormone (TSH) stimulate MCT1 promoter activity [11]. The presence of MCT1, CD147, and LDH in mitochondria promotes lactate importation and oxidation in the mitochondrial reticulum as well as supports the presence of a mitochondrial lactate oxidation complex[12].

### Lactate Receptor Including GPR81

Moreover, the lactate receptor *GPR81*, a G protein-coupled receptor regulates the trafficking of lactate between the plasma membrane and intracellular compartment, which suppresses cAMP and protein kinase A (PKA)-mediated signaling to inhibit lipolysis primarily in adipocytes [9]. *GPR81* is activated to quickly respond to high plasma lactate during intense exercise. Nonetheless, physiological concentrations of lactate are sufficient to activate *GPR81* [9, 13]. Madaan et al. revealed that lactate attenuates inflammation and ensuing preterm birth during labor by activating *GPR81* in the uterus [14]. Moreover, cancer cell–generated lactate activates *GPR81* to promote angiogenesis, immune evasion, and chemoresistance in tumors, thereby promoting the potential development of novel anticancer therapeutics [15]. Although it remains unclear whether *GPR81* is expressed on the mitochondrial membrane, *GPR81* knockdown causes a significant reduction in mitochondrial activity and a significant increase in cell death [15]. Lactate is transported across the cell nuclear membrane through simple diffusion and regulated by lactate concentration gradients.

### Histone Modifications

**Fig. 1:**
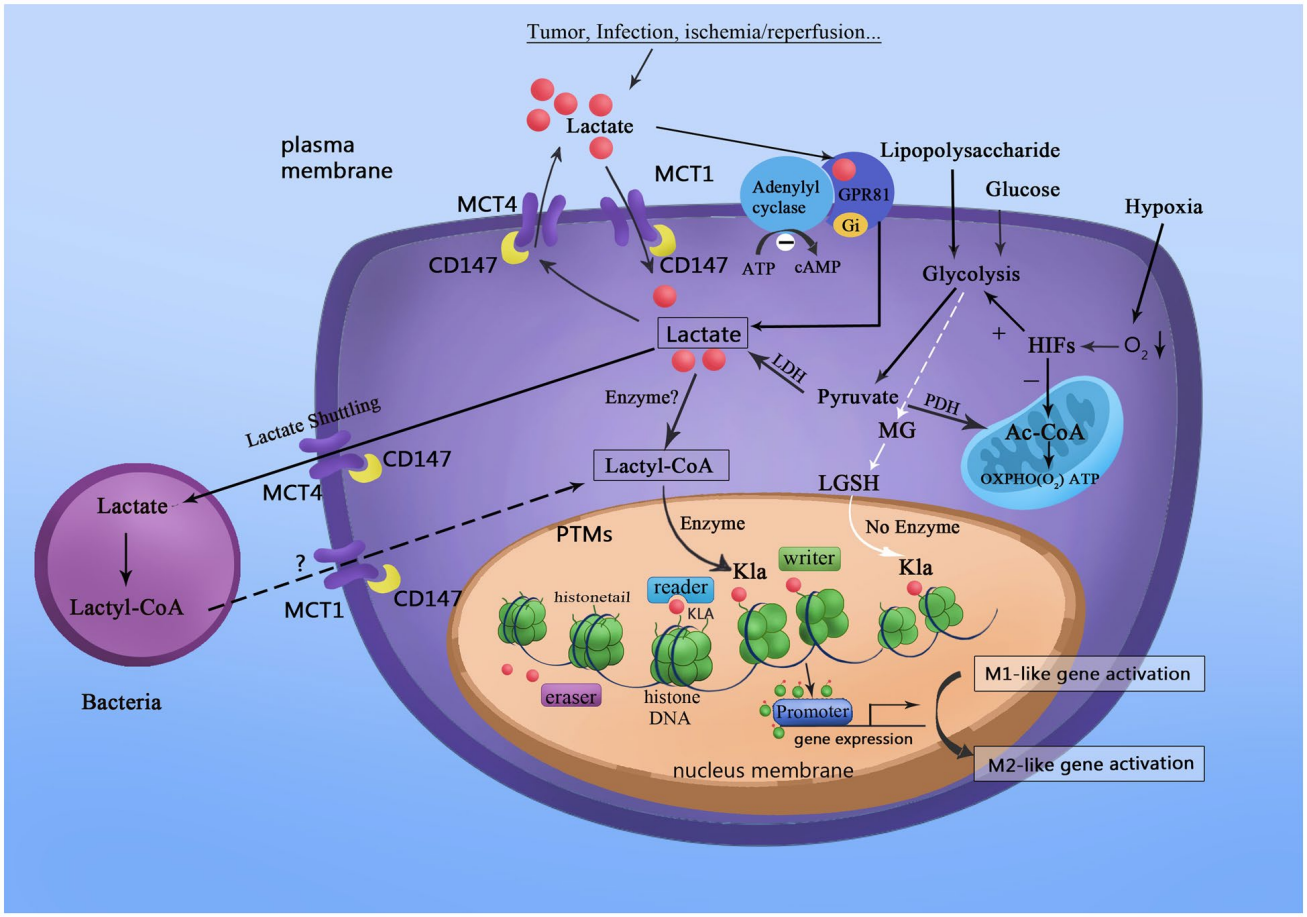
Lactate acts as a signaling molecule to stimulate gene transcription via histone lysine lactylation in M1 macrophages. Tumors, infection, ischemia, or reperfusion promote the movement of lactate into macrophages. Moreover, HIFs stimulate an increase of lactate by upregulating glycolysis and suppressing the Krebs cycle under hypoxic conditions. Nonetheless, lactyl-CoA synthetase or transferase activating lactate to lactyl-CoA remains unconfirmed. Microbiota contains enzymatic machinery that might convert lactate to lactyl-CoA with the help of lactyl-CoA synthetase. In return, lactyl-CoA stimulates histone Kla at the promoter DNA sequence, regulated by writer, eraser, and reader enzymes. Besides the enzyme-dependent transfer of the lactyl group from lactyl-CoA to lysine residues, non-enzymatic lactyl group transfer from LGSH to protein lysine residues generate a “LactoylLys” modification of proteins. Histone Kla is high in gene promoter regions for upregulating gene transcription, thereby promoting homeostatic M2-like polarization in M1 macrophages.

Histones are unique compounds comprising proteins (called the nucleosome core) and DNA in chromatin; their various posttranslational modifications (PTMs) regulate gene expression. Classical PTMs, including acetylation, malonylation, and succinylation, disrupt the spatial structure of proteins to regulate numerous processes in cellular physiology and biochemistry. Malonylation is an evolutionarily conserved post-translational modification (PTM) promoted by malonyl-CoA to add a malonyl group to lysine residues and change its charge from + 1 to –1 [16]. *SIRT5* regulates malonylation as a demalonylase and removes succinylation and glutarylation [17, 18]. Moreover, GAPDH and mammalian target of rapamycin (mTOR) undergo malonylation during macrophage activation, which regulates the production of proinflammatory cytokines [19]. Succinate accumulation promotes succinylation, which transfers a succinyl-CoA moiety to a lysine residue. In LPS-activated macrophages, succinylation of pyruvate kinase 2 at lysine residue K311 impairs glycolytic activity and induces nuclear translocation, where it promotes the transcription of hypoxia-inducible factor (HIF)-dependent genes and produces IL-1β [18, 20]. Different PTMs mediating glycometabolism is shown in Table 1. Histone lactylation is a novel form of PTM; lactate was first discovered by Zhang et al. as a signaling molecule that stimulates gene transcription via histone lysine lactylation in M1 macrophages [21] (Fig. 1). Also, Zhang et al. demonstrated that modulation of intracellular lactate production affects histone Kla levels in a dose-dependent manner; besides, they detected 13C-labeled atoms on histones by isotopically tracing the labeled glucose. Therefore, histone lactylation is a product of glycolysis and is regulated by lactate. Furthermore, intracellular metabolic perturbations are associated with the capacities of an epigenetic writer and eraser enzymes, which are regulated by histone acetylation and lactylation [4].

**Table 1:** PTMs in Glycometabolism

PTM	Metabolite	Residue modified	Protein targets	Function	References
Lactylation	Lactyl CoA	Lysine	Histones	Promotes M2-like polarization in M1 macrophages	[21]
			HMGB1	Induces vascular endothelial cell injury	[22]
Acetylation	Acetyl CoA	Lysine	Histones cGAS	Prevents self-DNA induced autoimmunity	[23]
			NLRP3	Activates macrophages	[24]
			GAPDH	Drives glycolysis and boosts rapid memory CD8+ T cell response	[25]
			HMGB1	Induces vascular endothelial cell injury	[22]
Malonylation	Malonyl CoA	Lysine	GAPDH	Controls the production of proinflammatory cytokines	[16]
			mTOR	Controls the production of proinflammatory cytokines	[26]
Succinylation	Succinyl CoA	Lysine	PKM2	Promotes the transcription of HIF-dependent genes and produces IL-1β	[18, 20]

### Non-enzymatic Lysine Lactylation

In addition to the enzyme-dependent transfer of lactyl group from lactyl coenzyme A (lactyl-CoA) to lysine residues, lysine acylation derived from the non-enzymatic reaction with lactoylglutathione (LGSH) has been demonstrated [27, 28]. Methylglyoxal, a glycolytic by-product, binds to glutathione via thioesterase glyoxalase I (GLO1) and generate LGSH, which is hydrolyzed to glutathione and D-lactate by GLO2. Gaffney et al. recently found that a non-enzymatic lactyl group transfer from LGSH to protein lysine residues generates a “LactoylLys” modification in proteins, which can be inhibited by GLO2 and regulate glycolytic in return [27]. Moreover, in mitochondrial proteins, non-enzymatic lysine acetylation by acetyl-CoA and acetylglutathione is promoted via a proximal S-acetylated thiol intermediate, which can be inhibited by GLO2 [29].

### Lactyl-CoA in Physiological and Pathological Conditions

Enzymatic lysine lactylation depends on both the presence of lysine acetyltransferase (KAT) enzymatic P300 and lactyl-CoA [21]. Nevertheless, a lactyl-CoA synthetase or transferase that activates lactate to lactyl-CoA in mammalian biochemistry has not been confirmed. Varner et al. recently showed that lactyl-CoA is quantifiable at 0.011 pmol 10–6 cells in HepG2 cells and 0.0172 pmol mg-1 tissue wet weight in the mouse heart; this is significantly less abundant (27–335 times lower) than other actyl-CoAs, including acetyl-, succinyl-, and propionyl-CoA [28]. Thus, a lower abundance of lactyl-CoA might limit the enzymatic lysine lactylation rate more than the KAT enzymatic P300. The modulation of lactyl-CoA under several important physiological conditions, including cancer and immune cell activation, makes lactylation an exciting signaling link between metabolism and cell signaling; however, additional research into its biochemistry and physiological roles are necessary [30, 31]. Microbiota such as *Escherichia coli* contains enzymatic machinery that may convert lactate to lactyl-CoA with the help of lactyl-CoA synthetase [32]. Zhang et al. found five efficient lactate CoA-transferases and a selective basis for the conversion of lactate into lactyl-CoA from *Clostridium propionicum* and *Megasphaera elsdenii*, which are important for regulating physiological and/or pathological conditions [33]. In the TME, the availability of lactyl-CoA may be linked to the nature of microbiotas equipped with lactyl-CoA synthetase [32]. Additionally, dysbacteriosis may promote lactyl-CoA production, which subsequently causes M2 macrophage polarization in sepsis [21]. Therefore, lactate and lactyl-CoA may shuttle between cancer cells, microbiota, and immune cells. Increased lactate production drives malignant cells toward excessive biomass production to fuel uncontrolled cell growth, a phenomenon known as the Warburg effect. Lactyl-CoA and acetyl-CoA levels potentially reflect histone lactylation and acetylation levels, the ratio of which marks the outlet of pyruvate and determines cell fate toward malignancy [34].

#### Upstream and Downstream Mechanisms of Histone Lactylation

Since it was first discovered by Vidali et al. in the 1960s, lysine acetylation (Kac) is an important modification of chromatin structure and modulator of gene transcription through histone regulation [35]. HIFs regulate various physiological processes by targeting transcription and translation during hypoxia, characterized by lactate production [36]. Under hypoxic conditions, HIFs stimulate the increase of lactate by upregulating glycolysis and suppressing the citric acid cycle [36]. However, whether HIFs also undergo lactylation under hypoxic conditions remains to be uncovered. Previous studies indicate that the acetylation of hypoxia-inducible factor-1α (HIF-1α) by *ARD1* improves the interaction of HIF-1α with von Hippel-Lindau tumor suppressor gene product (pVHL) and HIF-1α ubiquitination, which destabilize HIF-1α via proteasomal degradation [37]. Under normoxic conditions, HIF-1α binds to pVHL via hydroxylation of prolines. During hypoxia, HIF-1α phosphorylation triggers HIF-1 activation. Nevertheless, whether HIF-1α lactylation would induce destabilization remains unclear. Homeostasis of HIF-1α is regulated by these PTMs, including lactylation, acetylation, hydroxylation, ubiquitination, and phosphorylation. Lactylation might coordinate with other PTMs to maintain the homeostasis of HIF-1α. Zhang et al. identified 26 and 16 histone Kla sites in human HeLa cells and mouse bone marrow-derived macrophages (BMDMs), respectively [21]. Furthermore, Gao et al. identified 273 Kla sites in 166 proteins by analyzing the global lysine lactylome using LC–MS/MS [38]. Hagihara et al. identified 63 lactylated proteins in the mouse brain and noted that increased lactate levels, induced by neural excitation and social defeat stress, promote histone Kla in brain cells [39]. Accumulating studies indicate that acetylation and lactylation also exist in diverse non-histone proteins [38, 40]. Nonetheless, the modified function of non-histone lactylation, including modulating gene transcription, DNA damage repair, cell division, signal transduction, protein folding, autophagy, and metabolism, needs further research. Yang et al. recently demonstrated that lactate promotes lactylation and acetylation of high mobility group protein B1 (HMGB1), and its release from macrophages via exosomes to induce vascular endothelial cell injury and aggravate sepsis [22].

However, the enzymes producing lactyl-CoA and mechanisms by which histone Kla is regulated by writer, eraser, and reader enzymes remain to be studied. Carlos et al. revealed that class I histone deacetylases (HDAC1‒3) are histone lysine delactylases *in vivo* [5]. Lactylation and delactylation influence various physiological and pathological processes via epigenetic regulation and other mechanisms. For instance, lysine lactylation (Kla) is high in gene promoter regions, which promotes the binding of related transcription factors and gene promoters to upregulate gene transcription. Moreover, these metabolite PTMs, regulated by lactylation and delactylation influence protein function by targeting protein localization, conformation, stability, or interactions with other proteins or by competing with other PTMs [26]. Using M1 macrophages, Zhang et al. demonstrated that histone lactylation has different temporal dynamics from acetylation [21]. Histone Kac reached a peak at 3 h and a steady-state at 6 h, whereas histone Kla increased over a 24-h period [21]. ChIP-seq data obtained by Zhang et al. revealed that increased *H3K18la* marked more genes than decreased *H3K18la*, whereas *H3K18ac* was reversed [21]. In addition, most genes marked by increased *H3K18la* were specific, because 68% of these genes did not display significantly increased *H3K18ac* [21]. However, *H3K18ac*-specific genes remain to be identified [21]. Gene Ontology analysis revealed that these *H3K18la*-specific genes are enriched in biological pathway independent of inflammation, including Arginase 1 (*ARG1*) [21]. Additionally, a positive correlation has been observed between *ARG1* expression and histone Kla levels, but not histone Kac levels in tumor-associated macrophages (TAMs) [21]. Lactate metabolism and epigenetic reprogramming are a result of maintaining cellular homeostasis and inducing cancer cells to acquire various behaviors, including progression and invasiveness. The involvement of lactylation in different diseases is illustrated in Table 2.

**Table 2:** Lactylation in Diseases

Disease	Enzyme regulation	Histone modification	Cell proliferation	Protein targets	Gene targets	References
SCC	SIRT6		TPCs	GSH		[41]
Ocular melanoma		Lysine		YTHDF2	m^6^A	[42]
Lung fibrosis		Lysine	Macrophage			[43]

It has been demonstrated that glycolysis can proliferate TPCs in SCC. However, the mechanism of lactylation in SCC is unclear

#### The Function of Histone Lactylation in Cancer

Dysregulation of histone Kla by lactate disrupts the balance of gene transcription and causes diseases including cancer. Choi et al. recently discovered that the absence of NAD+-dependent histone deacetylase sirtuin 6 (SIRT6) enriches tumor-propagating cells (TPCs) by improving lactate production, in turn causing a much more aggressive tumorigenic phenotype in squamous cell carcinoma (SCC) [41]. TPCs regulate survival, recurrence, metastasis, therapeutic resistance, and immune resistance in various malignant tumors. Therefore, a hypothesis that *SIRT6* may regulate tumor proliferation by histone lactylation in TPCs warrants additional research. Another study demonstrated that histone Kla is essential in the oncogenic process. Yu et al. revealed that histone lactylation promotes tumorigenesis by facilitating the transcription of YTH N6-methyladenosine RNA-binding protein 2 (YTHDF2), which recognizes the m6A modification site in the mRNA of two tumor suppressor genes, *PER1* and *TP53*, and promotes their degradation in ocular melanoma [42]. For example, a consistently increased population of lactic acid bacteria in gastric cancer patients demonstrates that these bacteria potentially promote gastric cancer by supplying lactate and lactylation may be implicated in this process [44]. Moreover, tumor-related lactate induces robust production of alpha-ketoglutarate and is eventually associated with widespread epigenomic reprogramming in pancreatic ductal adenocarcinoma [45].

#### Stem Cell Regulation in Histone Lactylation

Lactate and histone lactylation significantly improve the survival, self-renewal, and reprogramming capacity of stem cells. High serum lactate dehydrogenase activating lactate to pyruvic acid, could be considered a predictor of poor patient survival in pre-and post-allogeneic hematopoietic stem cell transplantation [46]. Moreover, increasing evidence indicates that hypoxic preconditioning improves the survival of stem cells [47, 48]. Yang et al. revealed that leptin improves mitochondrial fusion-mediated glycolysis via sodium-glucose symporter 1-activated optic atrophy 1 to promote the survival of mesenchymal stem cell (MSC) [47]. Lee et al. further discovered that microbiota-derived lactate stimulates stem cell factor secretion by leptin receptor-expressing MSCs and subsequently activates hematopoiesis and erythropoiesis [49]. C-terminal binding proteins (CTBPs) are glycolytic sensors linking changes in metabolism to gene expression via NADH [48, 50]. CTBP dimerization promotes OCT4, SOX2, and NANOG expression to regulate the self-renewal of human embryonic stem cells (hESCs) [48]. Arthur et al. found that glycolysis regulates hESC self-renewal and CTBPs by modulating HIF-2α expression [48]. Recent studies indicate that glycolysis promotes stem cell-like properties in cancer cells. For instance, Yang et al. showed that the glycolytic enzyme enolase 1 could stimulate the stemness of gastric cancer cells to increase self-renewal, invasion, migration, and chemoresistance by enhancing glycolysis [51]. Also, Mamouni et al. found that the scaffold protein β-arrestin1 regulates the metabolic preference of stem cell-like bladder cancer cells toward glycolysis by inhibiting the mitochondrial pyruvate carrier MPC1 and promoting glucose transporter GLUT1 [52]. Histone lactylation and acetylation are vital processes linking metabolism and epigenetics. Gli-like transcription factor 1 upregulates glycolysis by opening glycolytic genes and closing somatic genes, which produce more lactate and acetyl-CoA [53]. Subsequently, improved acetylation and lactylation facilitate cellular reprogramming, which induces senescence in pluripotent stem cells [53].

#### Histone Lactylation Protects Against Inflammation and Promotes Tumor Growth via Macrophages

Macrophages protect against inflammation by remodeling tissue and removing cellular debris. They are divided into two subtypes, i.e., proinflammatory (termed M1) and anti-inflammatory (termed M2). Lactate directly inhibits signaling pathways and modifies histones to suppress inflammatory macrophage activation and promotes homeostatic M2-like polarization via several mechanisms at several subcellular locations [21, 54]. Different histone modifications implicated in the regulation of macrophage activation are shown in Table 3 [55]. Methylation and demethylation occur on the lysine and arginine residues of histones to regulate gene transcription, catalyzed primarily by lysine-specific demethylases and Jumonji C (JmjC) domain containing demethylases family demethylases [62]. Interferons (IFNs) significantly increase the host immunity for viral clearance and cancer immune surveillance. Moreover, lactate inhibits RLR signaling by binding the mitochondrial antiviral-signaling protein (MAVS) transmembrane domain and preventing MAVS aggregation, thereby promoting homeostatic macrophage polarization by indirectly suppressing type I IFN production [54, 63]. Macrophages detect bacterial pathogens through Toll-like receptors (TLRs), which induce early inflammation and pathogen elimination. Irizarry-Caro et al. recently demonstrated that the TLR signaling adapter B-cell adapter for PI3K (BCAP) inactivates glycogen synthase kinase 3β (GSK3β) and forkhead box protein O1 (FOXO1) and regulates the transition of macrophages from the inflammatory to the reparatory phenotype by promoting histone lactylation [64]. Zhang et al. discovered that histone Kla via the potential histone Kla writer protein p300 promotes the expression of M2-like genes in the late phase of M1 macrophage polarization after an inflammatory response to repair collateral damage [21]. Lactate-induced histone lactylation mediated by P300 promotes the expression of many profibrotic genes by macrophages in the lungs [43].

**Table 3:** Histone Modifications in the Regulation of Macrophage Activation

Histone modification	Function	Protein	References
Acetylation	Promotes M2 gene activation	P300	[56]
Deacetylation	Promotes M2 macrophage polarization	HDAC3	[57]
Deacetylation	Promotes insulin resistance via M1 macrophage polarization	SIRT6	[58]
Demethylation	Promotes M2 polarization via regulation of IRF4	JMJD3	[59]
Demethylation	Enhances M2 polarization	JMJD3	[60]
Demethylation	Induces M2 polarization in microglia through TrkA activation	JMJD3	[61]
Lactylation	Promotes homeostatic M2-like polarization	P300	[21]

Accumulating studies indicate that TAMs increase tumor progression in the highly lactate TME. Tumor-cell-derived lactate triggers the expression of vascular endothelial growth factor, *Arg1*, and the M2-like polarization of TAMs, mediated by HIF-1α, and promotes tumor growth [65]. Moreover, Liu et al. found that tumor-cell-derived lactate actively downregulates the expression of the macrophage lysosomal gene V-type proton ATPase subunit d2 (*ATP6V0D2*) via mTOR-dependent inhibition of TFEB, which subsequently targets HIF-2α for lysosome-mediated degradation [66]. The lactate-ATP6V0D2-HIF-2α axis causes improved tumor vascularization and growth [66]. Macrophages are crucial to various immune responses and are immunoregulatory cells within the tumor. Bohn et al. found an immune evasion mechanism where lactate-derived acidification causes macrophage G protein-coupled receptor-dependent expression of the transcriptional repressor ICER, which promotes TAM polarization toward a non-inflammatory phenotype and generates a permissive TME [67].

Additional studies on histone Kla are necessary to discover novel therapeutic targets and strategies for inflammation and tumors. Also, whether lactylation/acetylation/methylation ratio between different diseases or disease processes influence the prognosis remains unclear.

## UNSOLVED AND FUTURE DIRECTIONS

Lactate accumulating during metabolic processes is used as a precursor for histone Kla and participates in the regulation of M1 macrophage homeostasis, which in turn regulates inflammation, cancer, and other diseases. Nonetheless, these findings have raised many questions, and additional details warrant further research.

### General Questions

Zhang et al. reported that histone lactylation is cause by lactate accumulation and is sensitive to lactate levels [21]. However, whether histone lactylation is a necessary consequence of lactate accumulation, or a result of lactate analogs remains unclear. It remains unclear whether lactate concentration in the nucleus causes histone lactylation. Moreover, succination occurs at cysteine residues, and palmitoylation occurs at threonine residues [19]. It is also unclear whether lactylation occurs at amino acid residues other than lysine. Lactate is continuously produced in all types of cells in the body. Nonetheless, whether lactylation can be constitutively utilized in diverse cells, tissues, and organs in addition to under pathological conditions remains puzzling.

### Lactylation and Delactylation

Studies demonstrate that the nuclear receptor *Nur77* promotes signal transducer and activates transcription (STAT3) acetylation by recruiting P300 and reducing HDAC1 expression to improve the transcriptional activity of STAT3 via Nε acetylation [69]. In the nucleus, the transcriptional activity of STAT3 is inhibited by the interaction of lysyl oxidase-like 3 with STAT3 to deacetylate multiple acetyllysine sites [70]. Whether there is a specific writer or eraser for lactylation remains unclear. The factors influencing lactylation and delactylation may be regulated by the expression of P300, HDAC1, and lysyl oxidase-like 3. However, it remains unclear whether P300 catalyzes the transfer of lactyl from lactyl-CoA to histones *in vivo*.

### Lactylation in Gene Transcription

Acetylation causes chromatin decondensation, allowing access of transcription factors and other transcriptional coactivators to regions of gene transcription [68]. Histone acetylation neutralizes the positive charge of lysine residues, inducing chromatin remodeling to facilitate DNA access by the transcriptional machinery [68, 71]. Histone acetylation regulates the functions of transcription factors by improving their stability and transcriptional activity [72]. Whether similar mechanisms exist for lactate remains unclear. Moreover, mechanisms by which the cytoplasm and nucleus communicate to respond to changes in PTMs remain unclear.

### Lactylation in Treg Cells

Regulatory T (Treg) cells are vital in maintaining immune tolerance, controlling immune rejection, and preventing autoimmune diseases. Nonetheless, Treg cells help tumors evade the body immune surveillance and anticancer immunity. Accumulating studies indicate that lactate and glycolytic activity are essential regulators of Treg cell function in the tumor microenvironment [73, 74]. Tumors deprive effector T cells of nutrition to prevent destruction and provide lactate for Treg cells to resist effector T cells [73]. Zappasodi et al. demonstrated that glycolytic activity in tumors and Treg cells block cytotoxic T-lymphocyte-associated protein 4 (CTLA-4), an immune checkpoint of Treg cells and that glycolysis-low tumors might be more sensitive than glycolysis-high tumors to anti-CTLA-4 treatment [74]. Therefore, the function of histone Kla in regulating Treg cells warrants further study and maybe an essential reference point in the future.

### Drugs Targeting Histone Lactylation

Several drugs targeting histone PTMs have been clinically applied against various diseases and have achieved significant therapeutic effects. For instance, drugs decreasing histone methylation by inhibiting histone methyltransferases, those that decrease histone deacetylation by inhibiting HDACs, and the ones increasing histone crotonylation are beneficial to kidney injury [75]. Class I HDAC inhibitors (e.g., valproic acid) and activators influence many physiological processes mediated by lactylation and may be applied to treat diseases, similar to P300. Zhao et al. recently demonstrated that lactate promotes ferroptosis resistance in HCC tumor cells by activating the AMPK-SREBP1-SCD1 pathway, and whether lactylation that participates in this process remains unknown [76]. Taking control over the glycolytic switch from lactate production and lactylation to acetyl-CoA production and the TCA cycle may provide numerous opportunities to target cancer [34]. Therefore, comprehensive mechanisms of lactylation require additional research to identify more targets beneficial to drug research and development.

## CONCLUSION

For future clinical applications, it is crucial to understand the function and regulatory mechanism of histone lactylation in physiological and pathological processes highly dependent on glycolysis and lactate; these include tumor development, ischemic heart and brain disease, immune cell activation, and metabolic demands of anaerobic exercise. Generally, the discovery of histone lactylation provides a reference point for additional studies, including research on the site-specific functions of protein lactate modifications and the use of anti-lactate antibodies combined with LC–MS/MS techniques to study histone lactylation. Besides, histone lactylation could be beneficial for the in-depth study of many diseases and their processes.

## Data Availability

The data related to WB data used to support the findings of this study are available from the corresponding author upon request.
